# A longitudinal comparison of drug use among 10-year-old children and 15-year-old adolescents from the German GINIplus and LISAplus birth cohorts

**DOI:** 10.1007/s00228-015-1977-x

**Published:** 2015-11-19

**Authors:** Salvatore Italia, Irene Brüske, Joachim Heinrich, Dietrich Berdel, Andrea von Berg, Irina Lehmann, Marie Standl, Silke B. Wolfenstetter

**Affiliations:** Department of International Health, School for Public Health and Primary Care (CAPHRI), Faculty of Health, Medicine and Life Sciences, Maastricht University, Duboisdomein 30, 6229 GT Maastricht, The Netherlands; Helmholtz Zentrum München, German Research Center for Environmental Health, Institute of Epidemiology I, Neuherberg, Germany; Research Institute, Department of Pediatrics, Marien-Hospital Wesel, Pastor-Janßen-Str. 8-38, 46483 Wesel, Germany; Department of Environmental Immunology, UFZ – Helmholtz Centre for Environmental Research Leipzig, Permoserstr. 15, 04318 Leipzig, Germany; Helmholtz Zentrum München, German Research Center for Environmental Health, Institute of Health Economics and Health Care Management, Ingolstädter Landstraße 1, 85764 Neuherberg, Germany

**Keywords:** Adolescent, Child, Drug utilization, Longitudinal study, Anti-inflammatory drugs, Germany

## Abstract

**Purpose:**

The purpose of this study was to compare longitudinal data on drug utilization between 10-year-old children and 15-year-old adolescents and to analyse the association of drug use at the age of 15 years with drug use at the age of 10 years.

**Methods:**

Based on the German GINIplus (German infant study on the Influence of Nutrition Intervention plus environmental and genetic influences on allergy development) and LISAplus (Influence of lifestyle factors on the immune system and allergies in East and West Germany plus the influence of traffic emissions and genetics) birth cohorts, data on drug utilization (past 4 weeks) were collected using a self-administered questionnaire for 3642 children (10-year follow-up) and 4677 adolescents (15-year follow-up). The drugs were classified by therapeutic categories (conventional drugs, homeopathic drugs, etc.) and by codes according to the anatomical therapeutic chemical (ATC) classification system. Associations of adolescents’ drug use with gender, study area, maternal education, parental income, presence of chronic conditions, and prior drug use at the age of 10 years were analysed using a logistic regression model.

**Results:**

The 4-week prevalence rates of overall drug use were similar for adolescents (41.1 %) and children (42.3 %). However, adolescents used noticeably more anti-inflammatory drugs, analgesics, and systemic antihistamines. Exactly 3194 children/adolescents participated in both follow-ups. Adolescents’ use of anti-inflammatory drugs was predicted (OR = 3.37) by use of anti-inflammatory drugs as a child. In summary, the strongest predictor of adolescents’ use of specific therapeutic categories or ATC groups was the previous use of the same therapeutic drug category or ATC group as a 10-year-old child.

**Conclusions:**

Despite similar prevalence rates of overall drug utilization among both age groups, there is a noticeable difference concerning the use of drugs from specific ATC groups. Drug use as a child may partly determine what they use as an adolescent.

**Electronic supplementary material:**

The online version of this article (doi:10.1007/s00228-015-1977-x) contains supplementary material, which is available to authorized users.

## Introduction

Pharmaceutical products play an important role in the health system, both from the medical point of view as well as in economic terms. In Germany, the turnover in medicinal products (prescription drugs and self-medicated drugs together) amounted to about 34 billion euros in 2013 [1]. At about 30 billion euros, expenditures on medicinal products accounted for about 16 % of the total expenditures covered by the German statutory health insurance companies. They ranked third among their expenditure items in 2013 [1], only exceeded by costs of hospital treatments (65 billion euros) and medical expenses (32 billion euros). According to available publications, the prevalence of drug use is generally quite high among adolescents in Germany and other European countries as well. In Germany, a 1-week prevalence for overall drug use (prescription drugs and self-medicated drugs together) of 50.7 % was reported for adolescents aged 14–17 years [2]. Another German study that included both prescription drugs and self-medicated drugs found that 41 % of 15-year-old adolescents used at least one drug within an observation period of 4 weeks [3]. A Spanish study yielded similar results, with 31.6 % of children older than 10 years using prescription drugs or over-the-counter (OTC) drugs during the last 2 weeks prior to the assessment [4]. In other studies from Denmark, Italy, and the Netherlands that analysed prescription drug use only, the 1-year prevalence ranged between 38 and 57 % [5–9]. Moreover, the share of self-medicated drugs among all drugs used by adolescents may be considerable (e.g. reported at 69 % among 15-year-old adolescents and 38.5 % among children aged 0–17 years, respectively, from two German studies [2, 10]). Further European studies yielded prevalence rates for OTC drug or self-medicated drug use during childhood or adolescence of 17–39 % (use within the last 2 days) in Finland [11, 12], about 35 % (2-week prevalence) in the Netherlands [13], and 67 % (1-year prevalence) in Sweden [14]. The evidently high prevalence of (self-medicated) drug use among the general adolescent population raises some public health questions, e.g. whether all of the drugs taken are medically advisable and appropriate, or whether some self-medicated drugs such as analgesics are considered by adolescents to be ordinary commodities that may become part of everyday life over time.

The aim of this study is to analyse drug utilization longitudinally among children from two German birth cohorts by comparing drug use at two defined time points (at the age of 10 and 15 years, respectively). A further objective of this study was to detect whether there was an association of drug use at the age of 15 years with prior drug use at the age of 10 years.

## Methods

### Study population

The GINIplus study (German infant study on the Influence of Nutrition Intervention plus environmental and genetic influences on allergy development) and the LISAplus study (Influence of lifestyle factors on the immune system and allergies in East and West Germany plus the influence of traffic emissions and genetics) are based on two German birth cohorts [15, 16] that started with 5991 (GINIplus) and 3097 (LISAplus) healthy full-term newborns who were recruited between September 1995 and January 1999 from obstetric clinics in four German regions (Munich, Leipzig, Bad Honnef, Wesel). The recruitments sites were chosen to achieve an almost heterogeneous sample with regard to geographic region (South Germany: Munich; East Germany: Leipzig; West Germany: Bad Honnef and Wesel) and degree of urbanization (urban areas: Munich and Leipzig; comparatively rural areas: Bad Honnef and Wesel). From both studies, non-term children and children of less than 2500 g birth weight were excluded. Children with non-German parents or parents born outside Germany were not enrolled for the LISAplus study. Additionally, participants with insufficient German language skills were not eligible for both cohorts.

### Data collection

For the 15-year follow-up, exactly 6094 participants’ parents or legal guardians were contacted between January 2011 and October 2014. Socioeconomic variables such as parental education and income were collected with the main questionnaire, which also assessed the participants’ gender. Additionally, a separate self-administered questionnaire on drug utilization within the past 4 weeks assessed various details on the drugs that had been used by the adolescents within the past 4 weeks. The participants were asked to enter the drug names and the pharmaceutical identification numbers (PZN) into five designated spaces or to enclose the empty drug packages in a self-addressed envelope. The PZN, which is printed on the drug package, exactly identifies the drugs with respect to package size, dosage, manufacturer, listed price, etc. In case the limited number of designated spaces was not sufficient to enter all drugs, the participants were invited to separately note the precise number of drugs used. An almost similar methodology was adopted for the 10-year follow-up, in which 6541 parents/legal guardians had been contacted between October 2006 and October 2009. Exactly 3194 subjects participated in the 10-year follow-up and the 15-year follow-up as well.

### Drug classification

According to the German Medicines Act, homeopathic, anthroposophic, and herbal products are defined as medicinal products as well. Therefore, all reported drugs were considered for analysis and were classified into several therapeutic modalities such as conventional drugs with chemical active pharmaceutical ingredients, homeopathic drugs, and herbal drugs. The exact definition of the various therapeutic modalities has been described in detail in a previously published study [17]. If available, codes were assigned to the drugs according to the anatomical therapeutic chemical (ATC) classification system [18, 19].

### Outcome definition and statistical analysis

Participants who reported use of at least one drug from any therapeutic category within the past 4 weeks were classified as ‘overall drug users’. Those participants taking one or more drugs from a specific drug category (e.g. homeopathic drugs) or ATC group (e.g. ATC *N02* for ‘analgesics’) were classified as users of the respective drug category (e.g. ‘homeopathy users’) or ATC group (e.g. ‘analgesic users’).

For analysis, the statistical software package SAS was used (SAS Institute Inc., Cary, NC, USA, version 9.3). Bivariate associations were tested with the Pearson chi^2^ test (*p* < 0.05). Interaction between the independent variables was checked using Pearson’s correlation coefficients. Odds ratios (ORs) and their 95 % confidence intervals (CIs) were obtained from a multivariate logistic regression model that included several independent variables (study area, child’s gender, maternal education, parental income, presence of a child’s chronic disease, previous use of the same defined drug categories or ATC groups at the age of 10 years). In addition, for all models, a stepwise backward elimination procedure was performed at a significance level for removal from the model with *p* ≥ 0.05. Associations with *p* < 0.05 were considered to be significant. Mothers rather than fathers may decide on the (self-) medication of their children. Thus, for the definition of educational status, the mothers’ educational background was used. To account for differences in school systems between West and East Germany before reunification in 1990, maternal education was classified into three levels based on the mothers’ maximum completed years of schooling:

- Level 1: low education level (<10 years)
- Level 2: medium education level (exactly 10 years)
- Level 3: high education level (>10 years)

Mothers who did not report any school degree at all (*n* = 26) were allocated to the low education level. Entries for mothers (*n* = 14) reporting another (but not further specified) kind of school degree than those listed above were treated as missing values for educational status.

Income status was classified following the median equivalent income (MEI) of 2012 (€1,633 net/month for the 15-year follow-up) and 2008 (€1,549 net/month for the 10-year follow-up) [20, 21], where the household members were weighted according to the new scale of the Organisation for Economic Co-operation and Development (OECD) [22]. Three income levels were defined: low (≤60 % of MEI), medium (60–100 % of MEI), and high (>100 % of MEI). The income cutoffs correspond to the definition of poverty (60 % of MEI) [23].

To take into account also the impact of chronic disorders on drug use, adolescents were defined as chronically ill if the participants reported the diagnosis of a chronic disease by a physician during the preceding 5 years. The following chronic conditions (physician diagnosed) were considered: hay fever, perennial allergic rhinitis, food allergy, atopic dermatitis, and asthma. Additionally, also further self-reported chronic conditions such as diabetes, and celiac disease were considered.

The GINIplus and LISAplus cohorts obtained approval from the ethics committees of the Bavarian Medical Council, the University of Leipzig, and the Medical Council of North Rhine-Westphalia. Furthermore, written informed consent was given by the participants’ parents or legal guardians and by participants.

## Results

The questionnaires on drug utilization were completed for 3642 children at the 10-year follow-up (response rate 55.7 %) and 4677 adolescents at the 15-year follow-up (response rate 76.8 %). Compared with baseline, both samples showed no variation with regard to gender (*p* = 0.9936 (10-year follow-up); *p* = 0.4437 (15-year follow-up)), while the composition differed significantly with regard to study area (*p* < 0.0001 (10-year follow-up); *p* = 0.0023 (15-year follow-up)) and maternal education (both samples at *p* < 0.0001). The differences between the samples in the 10-year follow-up and the 15-year follow-up were not significant with regard to gender (*p* = 0.5375) and maternal education (*p* = 0.3696), whereas both samples varied significantly (*p* < 0.0001) with regard to study area and household income. The detailed composition of the cohorts over time is displayed in Table 1. The total number of reported drugs amounted to 3215 drugs in the 10-year follow-up and 3873 drugs in the 15-year follow-up.

**Table 1:** Characteristics of the GINIplus and LISAplus cohort over time

	Distribution of the strata in %			*p* value^b^
	Baseline	10-year follow-up	15-year follow-up	
Total number of participants	9088	3642	4677	
Gender				
Male	51.3	51.3	50.6	0.5375
Female	48.7	48.7	49.4	
Study area				
Munich	48.6	50.8	50.1	<0.0001
Leipzig	10.7	10.5	8.9	
Bad Honnef	3.4	5.4	4.0	
Wesel	37.3	33.3	37.0	
Maternal education				
Low	16.0	9.8	10.7	0.3696
Medium	39.0	39.4	38.6	
High	45.0	50.8	50.7	
Household income^a^				
≤60 % of MEI		16.8	17.0	<0.0001
60–100 % of MEI	NA	44.2	37.9	
>100 % of MEI		39.0	45.1	

*MEI* median equivalent income (MEI not available for the baseline survey), *NA* not available

^a^Based on the MEI of 2008 (€1,549; 10-year follow-up) and on the MEI of 2012 (€1,633; 15-year follow-up)

^b^Derived from chi^2^ test, testing the difference of the sample composition between the 15-year follow-up and the 10-year follow-up

In summary, there was no statistically significant difference (*p* = 0.2555) with regard to the prevalence rates of overall drug use between 15-year-old adolescents (41.1 %; 95 % CI 39.7–42.5) and 10-year-old children (42.3 %; 95 % CI 40.7–43.9). About 71 % of the drugs used by adolescents were OTC drugs (29 % were prescription drugs). The corresponding figures for 10-year-old children were 75 % (OTC drugs) and 25 % (prescription drugs). Compared with 10-year-old children, 15-year-old adolescents used fewer homeopathic and herbal drugs, whereas they took more conventional drugs containing chemical active ingredients (Supplementary Figure S1). The respective prevalence rates of use differed significantly between both age groups at *p* < 0.0001 (homeopathy use), *p* < 0.0001 (use of herbal drugs), and *p* = 0.0006 (use of conventional drugs). Overall, adolescents took fewer OTC drugs than children (*p* = 0.0077).

The 12 most frequent ATC codes in each follow-up, which accounted for 36.3 % (10-year follow-up) and 37.6 % (10-year follow-up) of all reported drugs in the corresponding follow-up, are displayed in Table 2. In the 10-year follow-up, one single homeopathic remedy (Arnica globules (*n* = 59), not listed in Table 2 as no ATC code available), was also among the most mentioned drugs. Four out of 12 of the most commonly used active ingredients that were taken by adolescents were anti-inflammatory agents (ibuprofen, naproxen) or analgesics (paracetamol, acetylsalicylic acid).

**Table 2:** Most frequent ATC codes (absolute number and percentage of all reported drugs)

10-year follow-up				15-year follow-up			
ATC	Active ingredient	*n*	%	ATC	Active ingredient	*n*	%
N02BE01	Paracetamol	191	5.9	M01AE01	Ibuprofen	484	12.5
M01AE01	Ibuprofen	169	5.3	N02BE01	Paracetamol	204	5.3
R01AA07	Xylometazoline	125	3.9	N06BA04	Methylphenidate	108	2.8
R03AC02	Salbutamol	114	3.5	R06AE07	Cetirizine	101	2.6
N06BA04	Methylphenidate	99	3.1	R01AA07	Xylometazoline	101	2.6
R05CB01	Acetylcysteine	91	2.8	R03AC02	Salbutamol	85	2.2
R05CB06	Ambroxol	76	2.4	R05CB01	Acetylcysteine	82	2.1
R01BP30	Systemic rhinologicals^a^	68	2.1	R01BP30	Systemic rhinologicals^a^	74	1.9
R05CP02	Ivy leaves	68	2.1	R05XH20	Homeopathic flu remedies^a^	59	1.5
R05XH20	Homeopathic flu remedies^a^	62	1.9	H03CA01	Iodide	56	1.4
R03BA02	Budesonide	54	1.7	N02BA01	Acetylsalicylic acid	54	1.4
R06AE07	Cetirizine	53	1.6	M01AE02	Naproxen	50	1.3

3215 drugs used at the 10-year follow-up and 3873 at the 15-year follow-up

*ATC* anatomical therapeutic chemical classification system

^a^Combined preparations

**Fig. 1:**
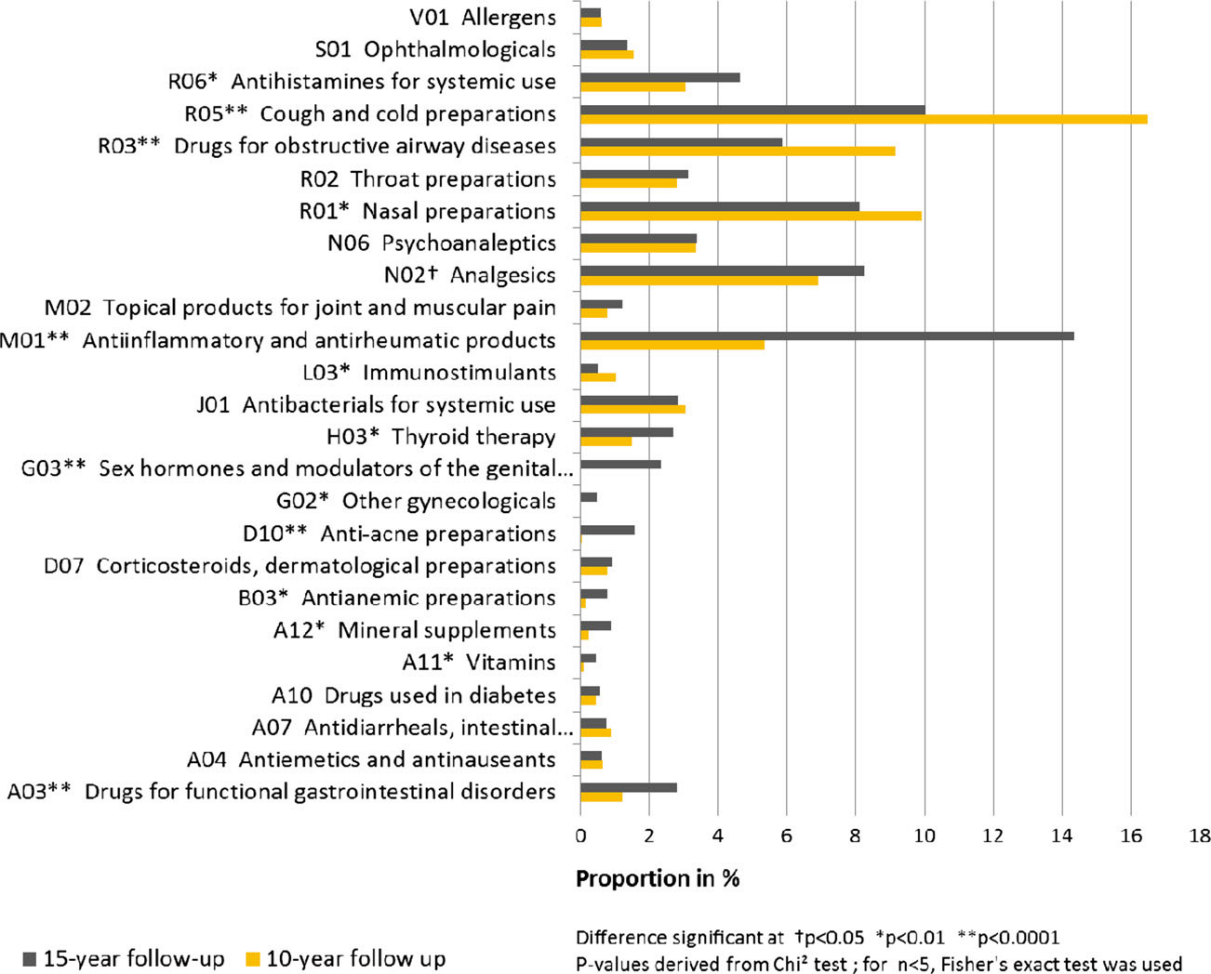
Comparison by ATC codes (only ATC groups with *n* > 15 in the 15-year follow-up considered, reflecting 79 % of all drugs used at the age of 15 years and 70 % of all drugs used at the age of 10 years). The displayed proportions refer to all reported drugs of the respective follow-up (*n* = 3215 drugs at 10-year follow-up and *n* = 3873 drugs at the 15-year follow-up)

The comparison by ATC group (Fig. 1) revealed that adolescents took fewer drugs from the ATC groups *R03* (drugs for obstructive airway diseases; *p* < 0.0001), *R05* (cough and cold preparations; *p* < 0.0001), and *R01* (nasal preparations; *p* = 0.0076). On the other hand, they used substantially more (*p* < 0.0001) anti-inflammatory drugs (ATC *M01*) and somewhat more analgesics (ATC *N02*; *p* = 0.0323), antihistamines (ATC *R06*; *p* < 0.0006), and drugs for functional gastrointestinal disorders (ATC *A03*; *p* < 0.0001).

**Fig. 2:**
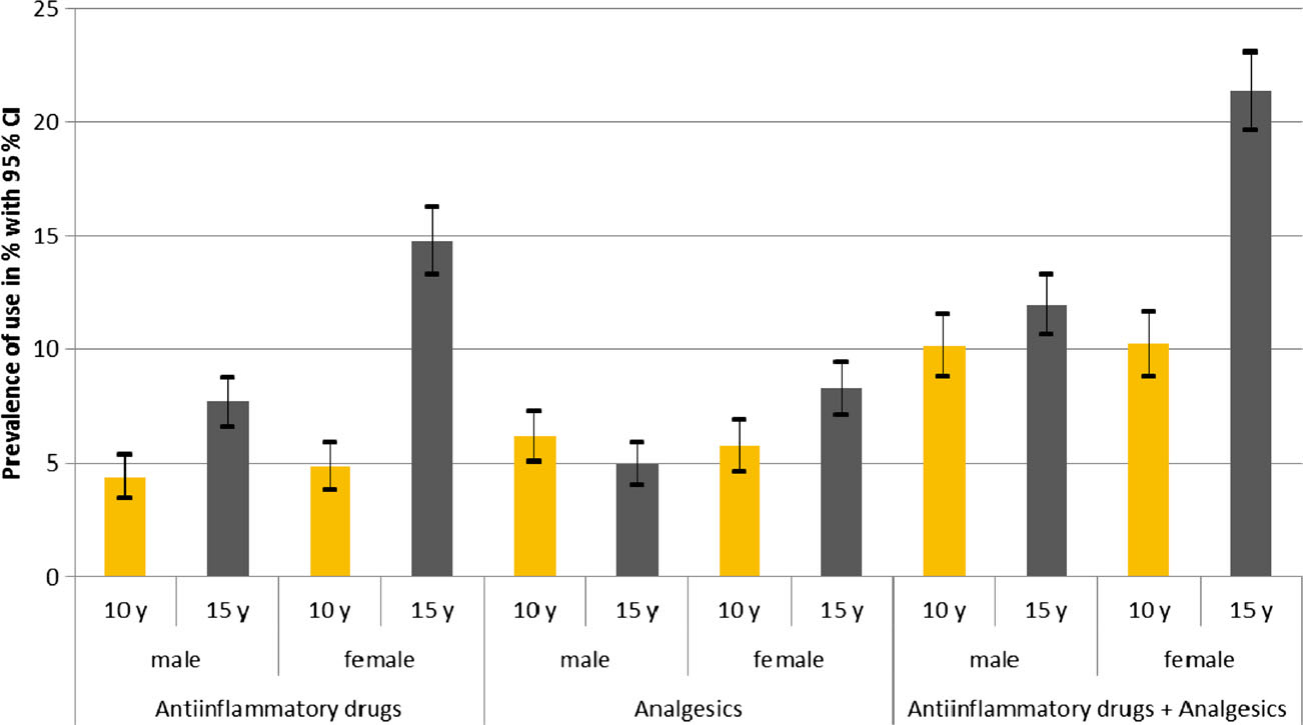
Prevalence of use of anti-inflammatory drugs and analgesics stratified by gender

A detailed view of the prevalence rates of anti-inflammatory drug use and analgesic use, which increased from 4.6 % (95 % CI 3.9–5.3) to 11.2 % (95 % CI 10.3–12.1) and from 6.0 % (95 % CI 5.2–6.7) to 6.6 % (95 % CI 5.9–7.3), respectively, showed that the higher prevalence rates among adolescents resulted mainly from an increase in the female stratum (Fig. 2), where 15-year-old girls used, for example, more than three times the number of anti-inflammatory drugs (4.9 % (95 % CI 3.8–5.9) vs. 14.8 % (95 % CI 13.3–16.2)). Additionally, there was no gender difference with regard to the use of anti-inflammatory drugs or analgesics among 10-year-old children, whereas 15-year-old girls used significantly more anti-inflammatory drugs or analgesics than 15-year-old boys (Fig. 2).

### Medication tracking

From the 3642 children who participated in the 10-year follow-up, exactly 3194 (87.7 %) were still in the GINIplus/LISAplus cohorts at the 15-year follow-up. Adolescents were more than twice as likely (OR = 2.25; 95 % CI 1.94–2.61) to be an ‘overall drug user’ if they had already been an ‘overall drug user’ at the age of 10 years. With regard to the use of the various drug categories (Table 3), the likelihood of being a drug user of the same drug category at the age of 10 years and as an adolescent as well was highest for homeopathic drugs (OR = 3.82; 95 % CI 2.91–5.00).

**Table 3:** Predictors of use of conventional drugs, homeopathic drugs, and herbal drugs (full models without backward elimination)

	Adjusted odds ratio of utilization (and 95 % confidence interval)					
	Conventional drugs		Homeopathic drugs		Herbal drugs	
Gender						
Male	Reference		Reference		Reference	
Female	1.54**	(1.32–1.79)	1.57*	(1.22–2.02)	1.33	(1.00–1.78)
Study area						
Munich	Reference		Reference		Reference	
Leipzig	0.91	(0.70–1.19)	1.10	(0.72–1.69)	1.50	(0.94–2.39)
Bad Honnef	1.25	(0.88–1.78)	1.11	(0.62–1.97)	1.48	(0.80–2.76)
Wesel	1.07	(0.89–1.28)	0.86	(0.64–1.17)	1.30	(0.92–1.83)
Maternal education						
Low	Reference		Reference		Reference	
Medium	*1.42*	(1.05–1.92)	*1.92*	(1.05–3.51)	1.77	(0.87–3.60)
High	*1.43*	(1.05–1.93)	*2.10*	(1.15–3.83)	*2.33*	(1.15–4.74)
Household income						
Low	Reference		Reference		Reference	
Medium	1.22	(0.95–1.57)	1.09	(0.70–1.69)	1.32	(0.82–2.12)
High	1.08	(0.83–1.40)	1.05	(0.67–1.64)	0.90	(0.55–1.49)
Chronic disease^a^						
No	Reference		Reference		Reference	
Yes	1.98**	(1.68–2.34)	*1.53*	(1.18–1.99)	1.20	(0.87–1.64)
Previous use of conventional drugs^b^						
No	Reference					
Yes	2.45**	(2.08–2.88)				
Previous use of homeopathic drugs^b^						
No			Reference			
Yes			3.82**	(2.91–5.00)		
Previous use of herbal drugs^b^						
No					Reference	
Yes					3.28**	(2.29–4.71)

Italicized numbers are significant at *p* < 0.05

*p < 0.01; **p < 0.0001

^a^Diagnosed by a physician during the preceding 5 years

^b^Use at the age of 10 years

The independent variables for previous use of conventional drugs, homeopathy, and herbal drugs, as displayed in Table 3, remained in the models after performing a backward elimination procedure. The longitudinal association of drug use was also given for various ATC groups. Table 4 shows the odds ratios of drug utilization from the most common ATC groups (except ATC group *G03*, where no use was reported among 10-year-old children), derived from the final multivariate logistic regression model with backward elimination.

**Table 4:** Odds ratios of drug use for various ATC groups (multivariate logistic regression model with backward elimination)

	ATC groups										
	A03	H03	J01	M01	N02	N06	R01	R02	R03	R05	R06
Gender (Ref=male)	8.56**	^b^	^b^	2.09**	1.71*	^b^	^b^	^b^	^b^	^b^	^b^
Study area (Ref=Munich)	^b^	^b^	^b^	^b^	^b^	^b^	^b^	^b^	^b^	^b^	^b^
Leipzig	^b^	^b^	^b^	*0.71* ^a^	^b^	^b^	^b^	^b^	^b^	^b^	^b^
Bad Honnef	^b^	^b^	^b^	*1.64*	^b^	^b^	^b^	^b^	^b^	^b^	^b^
Wesel	^b^	^b^	^b^	*0.77* ^a^	^b^	^b^	^b^	^b^	^b^	^b^	^b^
Maternal education (Ref=low)	^b^	^b^	^b^	^b^	^b^	^b^	^b^	^b^	^b^	^b^	^b^
Medium	^b^	^b^	^b^	^b^	^b^	^b^	^b^	^b^	^b^	^b^	^b^
High	^b^	^b^	^b^	^b^	^b^	^b^	^b^	^b^	^b^	^b^	^b^
Parental income (Ref=low)	^b^	^b^	^b^	^b^	^b^	^b^	^b^	^b^	^b^	^b^	^b^
Medium	^b^	0.32*	^b^	*0.97* ^a^	^b^	*0.56* ^a^	^b^	^b^	^b^	^b^	*1.23* ^a^
High	^b^	0.29*	^b^	*1.32* ^a^	^b^	0.28*	^b^	^b^	^b^	^b^	*1.87* ^a^
Chronic disease (Ref=no)	^b^	^b^	^b^	^b^	^b^	^b^	1.91**	^b^	8.71**	^b^	7.36**
Previous use of A03 (Ref=no)	^b^	^b^	^b^	^b^	^b^	^b^	^b^	^b^	^b^	^b^	^b^
Previous use of H03 (Ref=no)	^b^	78.97**	^b^	^b^	^b^	^b^	^b^	^b^	^b^	^b^	^b^
Previous use of J01 (Ref=no)	^b^	^b^	3.95*	^b^	^b^	^b^	^b^	^b^	3.82*	^b^	^b^
Previous use of M01 (Ref=no)	^b^	^b^	^b^	3.37**	^b^	^b^	2.11*	^b^	^b^	^b^	^b^
Previous use of N02 (Ref=no)	*2.50*	^b^	^b^	2.84**	3.25**	^b^	^b^	^b^	^b^	^b^	^b^
Previous use of N06 (Ref=no)	^b^	^b^	^b^	^b^	^b^	89.28**	^b^	^b^	^b^	^b^	^b^
Previous use of R01 (Ref=no)	^b^	^b^	^b^	^b^	^b^	^b^	2.54**	^b^	^b^	^b^	^b^
Previous use of R02 (Ref=no)	^b^	^b^	*2.83*	^b^	2.37*	^b^	^b^	3.74*	^b^	^b^	^b^
Previous use of R03 (Ref=no)	^b^	^b^	^b^	1.93*	^b^	^b^	^b^	^b^	17.71**	^b^	2.65*
Previous use of R05 (Ref=no)	^b^	^b^	^b^	*1.38*	^b^	^b^	^b^	^b^	^b^	2.21**	*1.91*
Previous use of R06 (Ref=no)	^b^	^b^	^b^	^b^	^b^	^b^	2.26*	^b^	^b^	^b^	5.38**

A03, drugs for functional gastrointestinal disorders

R01, nasal preparations

H03, thyroid therapy

R02, throat preparations

J01, antibacterials for systemic use

R03, drugs for obstructive airway diseases

M01, anti-inflammatory drugs

R05, cough and cold preparations

N02, analgesics

R06, antihistamines for systemic use

N06, psychoanaleptics

*Ref* reference

Point estimates in italics were significant at *p* < 0.05

*significant at *p* < 0.01

**significant at *p* < 0.0001

^a^Variable remained in the final model, but the calculated point estimate was not significant at *p* < 0.05

^b^Variable removed from the model by backward elimination

In most instances, the strongest predictor of adolescents’ drug use was the previous use of drugs from the same ATC group at the age of 10 years. For example, adolescents used significantly more antibacterials (OR = 3.95; 95 % CI 1.75–8.91), anti-inflammatory drugs (OR = 3.37; 95 % CI 2.28–4.99), analgesics (OR = 3.25; 95 % CI 2.13–4.94), or throat preparations (OR = 3.74; 95 % CI 1.67–8.38), if they had used drugs from the same ATC group 5 years before. The continuity of using the same type of drugs was visible for some very narrowly defined ATC subgroups as well, e.g. the use of ibuprofen (ATC *M01AE01*), paracetamol (ATC *N02BE01*), or mucolytics (*R05CB*) as an adolescent was predicted by the prior use of ibuprofen (OR = 3.71; 95 % CI 2.49–5.53), paracetamol (OR = 3.80; 95 % CI: 2.29–6.30), or mucolytics (OR = 3.67; 95 % CI: 1.88–7.16), respectively, at the age of 10 years. The model fit statistics for all regression models shown in Tables 3 and 4 were satisfactory (*p* at least *p* < 0.05). A detailed table that also contains the 95 % CI of the ORs shown in Table 4 is available as supplementary material to this article (Supplementary Table S2).

## Discussion

The findings of this study revealed that the prevalence of overall drug use was almost at the same level when comparing 15-year-old adolescents with 10-year-old children. At the same time, there was a noticeable difference in drug utilization with regard to specific ATC groups, e.g. a significantly higher use of anti-inflammatory drugs (ATC *M01*) among 15-year-old adolescents. The gender difference among 15-year-old adolescents may presumably in part result from the fact that female adolescents use e.g. ibuprofen for period pains. A high paediatric use of anti-inflammatory drugs or analgesics was also found in other European studies [2, 10, 14, 24–26]. Generally, the high prevalence of drug use raises the question of whether all the drugs used by adolescents were really medically necessary or advisable, not least in view of the fact that medicinal products such as anti-inflammatory drugs, analgesics, or antihistamines can also have severe side effects [27–29].

Another finding of this study was that adolescents used fewer homeopathic drugs and herbal drugs. Other studies analysing herbal drug use only [30] or various complementary and alternative medicine (CAM) modalities [31] also found lower prevalence rates for herbal drugs or homeopathy. This may be explained in part by the fact that OTC drugs (almost all homeopathic and herbal drugs were OTC drugs) are not normally reimbursed by German statutory insurance companies for children older than 12 years [32], but a lower acceptance of homeopathy or phytotherapy might also be a reason for the lower prevalence rates found for adolescents, as adolescents may begin to take their own decisions on self-medication with drugs. Moreover, the perception that homeopathic or herbal drugs are supposedly gentle remedies (one possible reason for their high use among children) may decrease or may play a less important role during transition to adolescence. A further striking outcome was that adolescents’ drug use from a specific ATC group was strongly predicted by the previous use of a drug from the same ATC group. This could have been expected for drugs that are used for the treatment of chronic or persistent conditions such as asthma, attention deficit and hyperactivity disorder, or thyroid disorders. On the other hand, this is a remarkable result for medicinal products (prescribed drugs as well as self-medicated drugs) that are supposedly used predominantly to treat an acute condition such as antibiotics, anti-inflammatory drugs, or analgesics. With regard to analgesics, another study [33] yielded a similar result, finding a strong association between headache medicine use at the age of 27 years and prior use of headache medicine at the age of 15 or 19 years.

An additional aspect that has to be considered is the difference between both age groups with regard to the proportion of prescribed drugs. While most (64 %) of the drugs used by 10-year-old children were prescribed by a physician (only 36 % were purchased on the participants’ own initiative), the share of prescribed drugs may be much lower among adolescents (this proportion was not directly assessed in the 15-year follow-up, but may be estimated at somewhat higher than 30 %, as exactly 29 % were prescription drugs and presumably only a few of the reported OTC drugs used by adolescents may have been prescribed or recommended by a physician [32]). This high share of OTC drugs emphasizes the importance of personal health literacy and the role of advice from a physician or a pharmacist with regard to adolescents’ self-medication with drugs.

This study has strengths and limitations as well. The data are based on the recently completed follow-ups of two large German birth cohorts. The data collection for the 15-year follow-up was performed almost evenly over the four seasons, with slight peaks in April and June, whereas data for the 10-year follow-up were collected mainly in the autumn (54 %). Nevertheless, the prevalence rate for overall drug use in the autumn was roughly at the same level as for all four seasons together (it was highest in spring and winter and lowest in summer). The difference between the distributions of data assessment over the year may also partly explain the different prevalence rates of use of seasonally sensitive ATC groups (e.g. cough and cold remedies, anti-allergy). Additionally, the different composition of both cohorts with regard to study area and household income may also have contributed to the different prevalence rates of drug use between both age groups. Moreover, other socioeconomic variables may also predict paediatric drug use, but could not be analysed in this study because of lacking availability (e.g. migration background). To our knowledge, the number of studies that analysed longitudinal data on paediatric drug use is very limited. Owing to the relatively short observation period of 4 weeks, the recall bias may have been minimized. On the other hand, underreporting may be likely for some types of drugs (e.g. contraceptives). Furthermore, categorization by the main ATC groups is relatively rough, as some ATC groups such as R05 include various types of chemical active ingredients. On the other hand, similar associations with prior drug use were found for very narrowly defined ATC groups as well. Defined daily doses could provide further important data for the comparison of drug use between both age groups, but were not considered in this study because for many of the reported drugs (e.g. homeopathic drugs or drug entries with an unclear package size), no defined daily doses were available.

In sum, both studies cannot be considered as being representative for the total German population due to arbitrarily selection of the four study areas, the exclusion criteria at baseline, and the selective follow-up. Therefore, the results can be projected to the general German adolescent population to a limited extent only, as the present cohort may not exactly reflect the German mean, e.g. with regard to maternal education level, parental income, or the adolescents’ general health status.

## Conclusions

Apparently, adolescents’ drug utilization depends partly on their drug use as children and may also determine what they will use in adulthood. It should be kept in mind that drugs are special products and not simple commodities. They should be utilized according to the need and not following habitual patterns only. In Europe, OTC drugs are also gradually becoming available outside pharmacies [34, 35] and are often subject to free advertising. It remains a difficult balancing act between free availability of medicinal products, adequate drug prices, and the appropriate use of self-medicated drugs. Overall, a good advice on the safe use of self-medication drugs might best be ensured by pharmacies [36, 37] rather than non-medical sale points such as supermarkets or petrol stations.

## Electronic supplementary material

ESM 1 (DOC 92 kb)

ESM 2 (XLS 41 kb)
