# Supplementary material for: A longitudinal comparison of drug use among 10-year-old children and 15-year-old adolescents from the German GINIplus and LISAplus birth cohorts
Source: Eur J Clin Pharmacol. 2015 Nov 19;72:301–10. doi: 10.1007/s00228-015-1977-x (PMC4751195; doi:10.1007/s00228-015-1977-x)
Supplement: Supplementary file 1 — (DOC 92 kb) [file 228_2015_1977_MOESM1_ESM.doc]

**
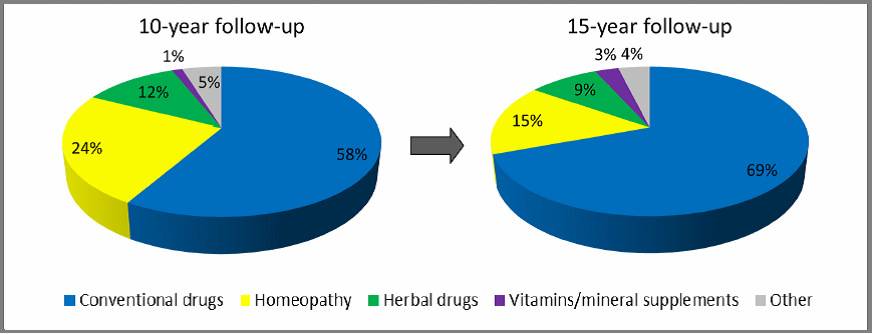
**

**Supplementary Fig. S1**

Comparison of drug utilization by drug category (preparations containing iodide, fluoride, and vitamin D used for prophylaxis according to medical guidelines were excluded from the category ‘Vitamins & mineral supplements’)
